# Cortisol and Inflammatory Biomarkers Predict Poor Treatment Response in First Episode Psychosis

**DOI:** 10.1093/schbul/sbv028

**Published:** 2015-03-31

**Authors:** Valeria Mondelli, Simone Ciufolini, Martino Belvederi Murri, Stefania Bonaccorso, Marta Di Forti, Annalisa Giordano, Tiago R. Marques, Patricia A. Zunszain, Craig Morgan, Robin M. Murray, Carmine M. Pariante, Paola Dazzan

**Affiliations:** ^1^Department of Psychological Medicine, King’s College London, Institute of Psychiatry, Psychology and Neuroscience, London, UK;; ^2^National Institute for Health Research Mental Health Biomedical Research Centre at South London and Maudsley NHS Foundation Trust and King’s College London, London, UK;; ^3^Department of Psychosis Studies, King’s College London, Institute of Psychiatry, Psychology and Neuroscience, London, UK;; ^4^Department of Health Services and Population Research, King’s College London, Institute of Psychiatry, Psychology and Neuroscience, London, UK

**Keywords:** HPA axis, cytokine, inflammation, outcome, stress, schizophrenia

## Abstract

*Background:* Cortisol and inflammatory markers have been increasingly reported as abnormal at psychosis onset. The main aim of our study was to investigate the ability of these biomarkers to predict treatment response at 12 weeks follow-up in first episode psychosis. *Methods:* In a longitudinal study, we collected saliva and blood samples in 68 first episode psychosis patients (and 57 controls) at baseline and assessed response to clinician-led antipsychotic treatment after 12 weeks. Moreover, we repeated biological measurements in 39 patients at the same time we assessed the response. Saliva samples were collected at multiple time points during the day to measure diurnal cortisol levels and cortisol awakening response (CAR); interleukin (IL)-1β, IL-2, IL-4, IL-6, IL-8, IL-10, tumor necrosis factor-α, and interferon-γ (IFN-γ) levels were analyzed from serum samples. Patients were divided into Non-Responders (*n* = 38) and Responders (*n* = 30) according to the Remission symptom criteria of the Schizophrenia Working Group Consensus. *Results:* At first onset, Non-Responders had markedly lower CAR (*d* = 0.6, *P* = .03) and higher IL-6 and IFN-γ levels (respectively, *d* = 1.0, *P* = .003 and *d* = 0.9, *P* = .02) when compared with Responders. After 12 weeks, Non-Responders show persistent lower CAR (*P* = .01), and higher IL-6 (*P* = .04) and IFN-γ (*P* = .05) when compared with Responders. Comparison with controls show that these abnormalities are present in both patients groups, but are more evident in Non-Responders. *Conclusions:* Cortisol and inflammatory biomarkers at the onset of psychosis should be considered as possible predictors of treatment response, as well as potential targets for the development of novel therapeutic agents.

## Introduction

Early treatment response is one of the strongest predictors of long-term symptomatic and functional outcome in psychosis.^1^ Unfortunately, we do not have reliable predictors of early treatment response in first episode psychosis, which makes it impossible to tailor psychiatric care to the needs of the individual patient. Biomarkers of stress and inflammation hold great potential as clinical predictors of treatment response: stress plays a recognized role in precipitating the onset and relapse of psychosis, and the cortisol stress response is already abnormal at psychosis onset^2–4^; moreover, increased inflammation has been shown to predict lack of a pharmacological response in depressed patients.^5,6^ In psychosis, neuroimaging biomarkers have been shown to predict treatment response, including our own work assessing cortical folding defects and white matter integrity in first episode psychosis,^7,8^ but neuroimaging findings may lack the immediate translational impact of a blood- or saliva-based biomarker. To our knowledge, no study has assessed whether (salivary) cortisol or serum inflammatory biomarkers predict treatment response at the onset of psychosis.

First episode psychosis patients show abnormalities in the activation of the main biological system involved in the stress response, the hypothalamic-pituitary-adrenal (HPA) axis.^9–11^ In particular, individuals at the onset of psychosis show a specific pattern of HPA axis abnormalities, distinct from depression or other psychiatric disorders, of increased cortisol levels throughout the day, blunted cortisol awakening response (CAR), and decreased cortisol response to psychosocial stressors.^12–14^ Interestingly, the blunted CAR and the reduced HPA axis reactivity to stress have also been associated with more severe symptoms and worse cognitive function in patients with psychosis.^15,16^ Furthermore, the blunted CAR is not normalized by antipsychotic treatment, indicating that it may represent a stable biological feature of psychosis.^12,17^

Recent work has also shown a role for inflammation in the pathogenesis of psychotic disorders.^18–20^ Individuals suffering with psychosis show increased cytokine levels in peripheral blood and cerebrospinal fluid and both at illness onset and in later stages of the disorder.^21^ We have also previously shown that increased inflammation and higher cortisol levels both contribute to smaller hippocampal volume at the onset of psychosis.^3^

Only few studies have attempted to clarify the association between stress or inflammatory biomarkers, and clinical outcome (not specifically treatment response), in psychosis. A previous study investigating HPA axis activity in patients with chronic schizophrenia has reported that persistent nonsuppression of cortisol levels following the dexamethasone test after 4 weeks of antipsychotic treatment was associated with poor clinical outcome.^22^ Conversely, a reduction in cortisol levels after 12 weeks of antipsychotic treatment was associated with an improvement in psychotic symptoms at 12 weeks follow-up, in both chronic and first episode psychosis patients.^23,24^ Only 3 studies investigated the link between inflammation and clinical outcomes in psychosis, and all were conducted in patients with chronic schizophrenia; interestingly, they all found that higher inflammation was associated with a poorer clinical outcome, as indicated by either less improvement or earlier relapses.^23,25,26^ Of note in this context, abnormal cortisol and inflammatory biomarkers have also been described in association with experiences of early life trauma, both in depression and in psychosis^12,18,27,28^; moreover, early life trauma in depression is associated with lack of treatment response,^29^ although no such data are available in psychosis.

We conducted a naturalistic longitudinal study in which first episode psychosis were assessed at baseline (ie, as soon as possible, and not later than 3 months, after the first contact with psychiatric services), and then were followed up prospectively for their treatment response at 12 weeks. The antipsychotic treatment was clinician-led, and we did not influence medication choice. We also completed a second biomarker assessment at 12 weeks. The aims of our study were: (1) to investigate whether cortisol and inflammatory biomarkers at *baseline* predicted treatment response at the 12 weeks follow-up; (2) to assess if *changes* in these biomarkers over the first 12 weeks were associated with treatment response in the same patients; and (3) to clarify if the putative relationships between these biomarkers and treatment response was partly influenced by previous experience of early life trauma.

## Methods

### Subjects Recruitment and Study Design

Sixty-eight first episode psychosis patients were recruited in South-East London (UK) as part of the Genetics and Psychosis study. The recruitment strategy was based on contacting inpatients and outpatients units of the South London and Maudsley (SLAM) NHS Foundation Trust, interviewing staff and reviewing clinical notes, and approaching all subjects aged 18–65 who presented for the first time to these services for a functional psychotic illness. Patients with organic psychosis, learning disabilities or not fluent in English were excluded from the study.^30,31^ Fifty-seven healthy controls were recruited from the same catchment area as the patients through advertisement in local newspapers, hospitals, and job centers, as well as from existing volunteer databases. Controls were screened using the Psychosis Screening Questionnaire^32^ and were excluded if they met criteria for a present or past psychotic disorder.

All patients were assessed as soon as possible after their first contact with psychiatric services, and not later than 3 months from this first contact. At 12 weeks, a clinical follow-up was completed on all patients to establish response, and a subset of 39 patients also repeated the biomarker measurements. Not all the 39 subjects completed both cortisol and cytokine assessments; in particular, of these 39 subjects, 24 repeated the cortisol assessment and 33 had serum collected and cytokines analyzed. The study was approved by the local Research Ethics Committee, in accordance with the code of ethics of the World Medical Association, and written informed consent was obtained from all participants.

### Clinical Assessment and Treatment Response

At the time of the first assessment, 7 patients were drug naive, 33 were taking olanzapine, 16 were taking risperidone, 4 were taking quetiapine, and 8 were taking aripiprazole. Thirty-seven patients received a DSM-IV diagnosis of schizophrenia/schizophreniform disorder, 22 of schizoaffective or affective psychosis, 7 of psychotic disorder not otherwise specified, and 2 of delusional disorder. Validation of clinical diagnosis was obtained using the Operational Criteria (OPCRIT+),^33^ reviewing the case notes in the first month following first contact with services. All diagnoses were performed by qualified psychiatrists, subject to comprehensive training and inter-rater reliability testing (κ = 0.9). Psychotic symptoms were evaluated both at baseline and follow-up, using the Positive and Negative Syndrome Scale (PANSS).^34^

Response to treatment at 12 weeks was evaluated using information obtained from clinical records, patient face-to-face interviews, and reports from informants, using the World Health Organization Personal and Psychiatric History Schedule—Follow-up, a standardized instrument to record presence and severity of symptoms that has been successfully used in World Health Organization multicenter studies of the incidence and outcome of schizophrenia.^35^ As we previously reported,^8^ response was operationalized as a reduction in symptom severity to the levels required by the remission criteria of the Schizophrenia Working Group Consensus.^36^ This consensus established a set of criteria that provide an absolute threshold in severity of symptoms that should be reached for clinical improvement. This approach was therefore preferred to symptom change cutoffs for this naturalistic study, since cut-off points are often arbitrary, affected by variability in baseline symptom severity across studies, and are not understood intuitively by clinicians. Instead, the remission criteria proposed by the consensus are more suited for traditional concepts of remission in psychiatric disorders. For those patients who could not be reassessed at 12 weeks (*n* = 20), information on treatment response was obtained using the Personal and Psychiatric History Schedule (PPHS).^35^ As previously published,^7,8^ for the purposes of treatment response, we considered the PPHS scores equivalent to the PANSS scores as follows: 0 was equivalent to PANSS scores 1, 2, and 3; 1 was equivalent to PANSS scores 5 and 6; and 2 was equivalent to PANSS scores 7 and 8. In a series of secondary analyses we also used a continuous measure of treatment response. This was estimated as change in PANSS total scores from baseline to follow-up, taking into account baseline PANSS total score and subtracting a score of 30, as even individuals without any mental health problem could score 30 in the PANSS. Therefore, as done in previous articles,^37^ we used the following formula: ((baseline PANSS total score − 30) − (follow-up PANSS total score − 30)/(baseline PANSS total score − 30) × 100).

We collected information about stressful life events that occurred in the previous 6 months using a brief life events questionnaire,^38^ and we measured perceived stress in the previous month using the Perceived Stress Scale.^39^ Information about childhood trauma was also collected using a modified version of the Childhood Experience of Care and Abuse Questionnaire, as previously published.^40^

The sociodemographic characteristics of the samples are shown in table 1. Using the above-mentioned criteria, 30 patients were classified as Responders and 38 as Non-Responders. Non-Responders had significantly higher scores of PANSS negative symptoms at baseline when compared with Responders (*P* = .03, see table 1). The mean duration of antipsychotic treatment at baseline was 35.5 ± 5.0 days for Responders and 46.3 ± 5.3 days for Non-Responders (*P* = .2). The cumulative dose of antipsychotic treatment received at the time of baseline assessment was not statistically different between Responders (chlorpromazine equivalents 8091.8 ± 1387.8) and Non-Responders (chlorpromazine equivalents 13 115.4 ± 2865.2, *P* = .2). The cumulative dose of antipsychotic treatment received at the time of follow-up assessment was also not significantly different between Responders (chlorpromazine equivalents 21 422.5 ± 3493.6) and Non-Responders (chlorpromazine equivalents 31 345.5 ± 5233.8, *P* = 0.1).

**Table 1.** Sociodemographic Characteristics and Baseline Levels of Stress Biomarkers

	Responders (*n* = 30)	Non-Responders (*n* = 38)	Controls (*n* = 57)	Test and Significance
Age (y)	29.4±1.4	29.1±1.3	26.8±0.6	*F* = 2.2, *df* = 2, 122, *P* = .1
Gender (M/F)	18/12	28/10	36/21	*χ* ^2^ = 1.7, *P* = .4
Ethnicity (white/others)	10/20	13/25	36/21	*χ* ^2^ = 10.7, ***P* = .005** ^a,b^
Number of recent stressful events	2.6±0.4	2.1±0.3	1.4±0.2	*F* = 5.2, *df* = 2, 117, ***P* = .007** ^a,b^
Perceived Stress Scale score	22.1±1.4	20.4±1.5	12.6±0.8	*F* = 21.5, *df* = 2, 118, ***P* < .001** ^a,b^
Childhood trauma (% with at least one trauma)	70%	84.2%	37%	*χ* ^2^ = 15.3, ***P* < .001** ^a,b^
Baseline PANSS positive symptoms	14.1±0.9	15.0±1.1	—	*t* = 0.6, *df* = 1, 65, *P* = .6
Baseline PANSS negative symptoms	13.7±0.9	16.8±1.0	—	*t* = 2.2, *df* = 1, 65, ***P* = .03**
Baseline PANSS general symptoms	29.2±1.3	29.4±1.1	—	*t* = 0.78, *df* = 1, 63, *P* = .9
Cortisol AUC-DAY (nmol h/l)	60.5±8.0	60.9±7.6	80.1±6.2	*F* = 8.0, *df* = 2,79, ***P* = .001** ^a,b^
Cortisol AUC CAR (nmol min/l)	705.7±72.7	506.8±63.0	910.4±55.3	*F* = 13.6, *df* = 2, 77, ***P* < .001** ^a,b,c^
IL1β (pg/ml)	1.8±0.4	3.0±0.8	2.5±0.4	*F* = 0.9, *df* = 2, 66, *P* = .4
IL2 (pg/ml)	3.2±0.8	2.1±0.5	3.3±0.4	*F* = 0.2, *df* = 2, 58, *P* = 0.8
IL4 (pg/ml)	4.5±0.5	6.0±1.1	3.7±0.2	*F* = 3.3, *df* = 2,60, ***P* = .04** ^b^
IL6 (pg/ml)	2.9±0.9	15.7±4.0	0.9±0.9	*F* = 19.0, *df* = 2,63, ***P* < .001** ^a,b,c^
IL8 (pg/ml)	200.1±45.5	171.3±47.6	44.3±9.6	*F* = 10.4, *df* = 2,61, ***P* < .001** ^a,b^
IL10 (pg/ml)	1.6±0.2	2.4±0.5	1.3±0.1	*F* = 3.5, *df* = 2,60, ***P* = .04** ^b^
TNFα (pg/ml)	13.3±2.3	16.0±1.7	6.4±0.5	*F* = 20.7, *df* = 2, 54, ***P* < .001** ^a,b^
IFNγ (pg/ml)	4.2±2.1	20.9±6.0	1.6±0.2	*F* = 12.1, *df* = 2, 62, ***P* < .001** ^a,b,c^

*Note*: AUC, area under the curve; CAR, cortisol awakening response; IFN, interferon; IL, interleukin; PANSS, Positive and Negative Syndrome Scale; TNF, tumor necrosis factor. *P* values <0.05 are in bold.

Post-hoc analyses significance is reported as follow:

^a^ *P* < .05 Responders vs Controls.

^b^ *P* < .05 Non-Responders vs Controls.

^c^ *P* < .05 Responders vs Non-Responders.

### Salivary Cortisol Assessment

Cortisol was measured in the saliva as CAR and daily profile in 65 patients and in 33 controls. Twenty-four of the 65 patients who completed cortisol assessments at baseline also completed cortisol assessment at 12 weeks follow-up. Subjects were instructed to collect saliva samples immediately after awakening (0min) and 15, 30, and 60min after awakening, and again at 1200h and at 2000h; details of saliva collection for these subjects have been already reported in previous articles.^12,16,41^ Cortisol levels were analyzed using the High Sensitivity Salivary Cortisol ELISA KIT from Salimetrics following the recommended procedure. As published before,^10^ a small number (20%) of samples were also measured using “Immulite”—DPC’s Immunoassay analyzer (www.diagnostics.siemens.com), and the reliability between the 2 methods was found to be very high (*z*-scores; *r* = .93; *P* < .001).

We used 2 summary measures of HPA axis activity: the area under the curve (AUC) of cortisol levels during the day (AUC-DAY; 0 min after awakening, 12 and 20 h) and the AUC of CAR (0, 15, 30, and 60 min after awakening); both formulas for the calculation of AUC were derived from the trapezoid formula as described by Pruessner et al^42^, and standardized *z* scores were used for statistical analyses, as previously published.^10^

### Cytokines Analyses

Blood samples were collected in 34 patients (at baseline) and 36 controls using clot activator tubes for serum analysis. All patients but one (*n* = 33) had serum collected and cytokines analyzed also at 12 weeks follow-up. The serum was separated, aliquoted, and stored at −70°C before use. Biochip array technology was used to perform simultaneous quantitative detection of multiple analytes from a single patient sample. The core technology is the Randox Biochip (http://www.randox.com), a solid-state device containing an array of discrete test regions of immobilized antibodies specific to different cytokines and growth factors. A sandwich chemiluminescent immunoassay was employed for the cytokine array. This cytokine array measures the following cytokines and growth factors: interleukin (IL)-1α, IL-1β, IL-2, IL-4, IL-6, IL-8, IL-10, tumor necrosis factor- α (TNF-α), interferon-γ (IFN-γ), vascular endothelial growth factor (VEGF), epidermal growth factor (EGF), and monocyte chemotactic protein-1 (MCP-1). Intra-assay precision and sensitivity of the cytokine array has been shown in previous published articles.^18^ We excluded IL-1α, VEGF, EGF, and MCP1 from the statistical analyses, as levels of most of the values were below the detection limit.

### Data Analyses

Data were analyzed using the Statistical Package for Social Sciences, Version 15.0 (SPSS Inc). Continuous variables are presented as mean ± standard error of the mean. Chi-square tests were used to compare categorical variables between patients and controls. One-way ANOVAs followed by least significant difference (LSD) post-hoc analyses were applied to test differences in biomarkers among Responders, Non-Responders, and controls at baseline. Repeated measures ANOVAs were applied to test longitudinal within-group changes (Responders and Non-Responders), and group × time (baseline and follow-up) interaction for those biomarkers that at baseline were significantly different between Responders and Non-Responders. Spearman’s correlations were performed to test the association between identified biomarkers of treatment response and percentage of clinical improvement. Boxplots of serum cytokines levels were generated to identify possible outliers for each separate cytokine; the identified outliers were removed before running statistical analyses. Serum cytokine levels were normalized for the statistical analyses through logarithmic transformation. Serum cytokines levels are presented as raw values, while the analyses were conducted using the logarithmic-transformed values.

## Results

### Baseline CAR and Inflammatory Biomarkers Predict Treatment Response in First Episode Psychosis

A one-way ANOVA, including Non-Responders (NR), Responders (R), and healthy controls (HC), revealed significant differences among the 3 groups for cortisol levels during the day (*P* = .001), CAR (*P* < .001), IL-4 (*P* = .04), IL-6 (*P* < .001), IL-8 (*P* < .001), IL-10 (*P* = .04), TNF-α (*P* < .001), and IFN-γ (*P* < .001). In contrast, no significant differences were found among the 3 groups for IL-1β and IL-2 (see table 1).

**Fig. 1.**
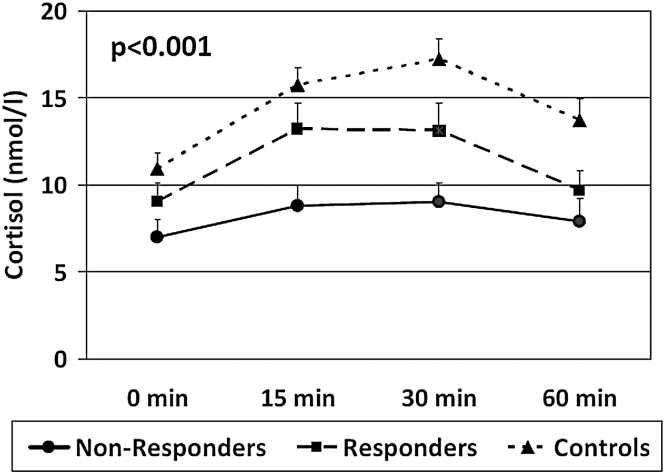
Baseline mean ± standard error of the mean of cortisol levels at 0, 15, 30, and 60min after awakening in Controls, Responders, and Non-Responders. *P* value indicates the significance of the comparison of the area under the curve of cortisol awakening response at one-way ANOVA.

**Fig. 2.**
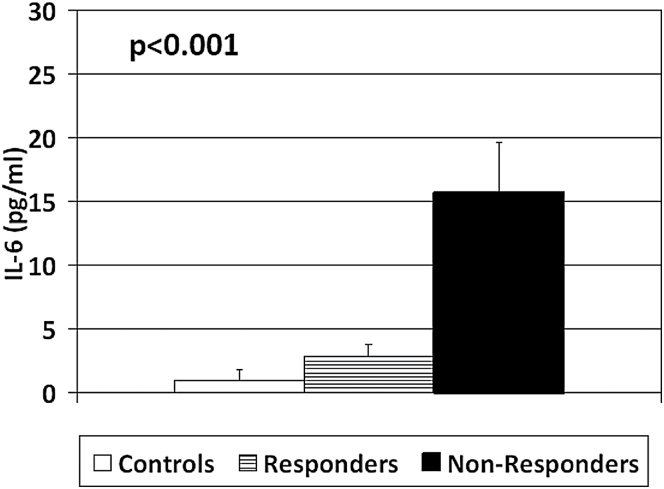
Baseline mean ± standard error of the mean interleukin (IL)-6 levels in Controls, Responders, and Non-Responders. *P* value indicates the significance of the comparison with the one-way ANOVA.

**Fig. 3.**
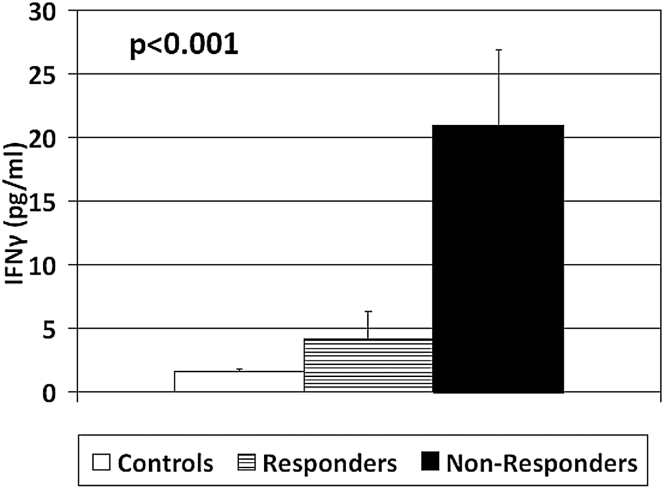
Baseline mean ± standard error of the mean interferon (IFN)-γ levels in Controls, Responders, and Non-Responders. *P* value indicates the significance of the comparison with the one-way ANOVA.

In the post-hoc analyses, only 3 variables differed between Non-Responders and Responders. In particular, Non-Responders showed a significantly lower CAR (*d* = −0.59, *P* = .03), and higher IL-6 (*d* = 1.05, *P* = .003) and IFN-γ levels (*d* = 0.88, *P* = .015) when compared with Responders (see figures 1–3). Of note, both Non-Responders and Responders had significantly lower CAR than healthy controls (respectively, *d* = −1.28, *P* < .001 and *d* = −0.62, *P* = .009), although the difference was greater in the Non-Responders, and the trend analysis revealed a significant linear relationship between CAR and group (*P* < .001: NR < R < HC, see figure 1). There was no significant difference in awakening time between the Responders and Non-Responders (*P* = .7).

As per CAR, both Non-Responders and Responders had significantly higher IL-6 levels than healthy controls (respectively, *d* = 1.24, *P* < .001; *d* = 0.75, *P* = .02), and the difference was again greater in the Non-Responders. The trend analysis revealed again a significant linear relationship between IL-6 and group (*P* < .001: NR > R > HC, see figure 2). Both Non-Responders and Responders also had higher levels of IFN-γ than healthy controls, although this difference was statistically significant only for the Non-Responders (*d* = 1.06, *P* < .001), while it reached a trend level of significance in the Responders group (*d* = 0.45, *P* = .09). The trend analysis revealed again a significant linear relationship between IFN-γ and group (*P* < .001: NR > R > HC, see figure 3).

For cortisol levels during the day, IL-4, IL-8, IL-10, and TNF-α, the post-hoc analyses showed no differences between Responders and Non-Responders; however, IL-4 and IL-10 were significantly higher only in Non-Responders when compared with controls, while they were no significantly different between Responders and controls. Moreover, cortisol levels during the day were lower, while IL-8 and TNF-α were higher, in both patients groups when compared with controls (see table 1).

### Longitudinal Changes in Cortisol and Inflammatory Markers

We evaluated changes over 12 weeks in Responders and Non-Responders for the markers that were significantly different at baseline between these 2 groups (ie, CAR, IL-6 and IFN-γ), with a repeated measures analysis (see table 2). We found a significant group effect for the CAR (partial η^2^ = 0.31, *P* = .01), IL-6 (partial η^2^ = 0.14, *P* = .04), and IFN-γ levels (partial η^2^ = 0.14, *P* = .05), indicating that Non-Responders, compared with Responders, maintained the lower CAR, as well as the higher IL-6 and IFN-γ levels, across the 2 time points. There was no time effect on either CAR (partial η^2^ = 0.05, *P* = .4) or IL-6 (partial η^2^ = 0.02, *P* = .4), with no group by time interaction (*P* = .9 and *P* = .7, respectively), indicating no significant changes in CAR and IL-6 levels across the 12 weeks in the 2 groups of patients. In contrast, there was a significant time effect on IFN-γ levels (partial η^2^ = 0.22, *P* = .009), with no group by time interaction (*P* = .6), indicating that IFN-γ levels increased with time in both samples of patients.

**Table 2.** Repeated Measures Analyses of Cortisol Awakening Response (CAR), Interleukin-6 (IL-6), and Interferon-γ (IFNγ) at Baseline and 3-mo Follow-up in Responders and Non-Responders

	Responders (*n* = 18)		Non-Responders (*n* = 21)		Test and Significance for Main Group Effect
	Baseline	Follow-up	Baseline	Follow-up	
Cortisol AUC CAR (nmol min/l)	766.1±147.6	589.2±153.4	513.8±72.6	314.9±70.0	*F* = 7.7, *df* = 1, 17, *P* = .01
IL6 (pg/ml)	3.0±1.0	16.7±10.7	16.1±4.2	34.6±12.6	*F* = 4.5, *df* = 1, 29, *P* = .04
IFNγ (pg/ml)	4.4±2.3	10.1±2.2	17.9±5.6	67.1±24.6	*F* = 4.4, *df* = 1, 28, *P* = .05

*Note*: Of the *n* = 39 patients assessed at follow-up, *n* = 24 (*n* = 12 Responders and *n* = 12 Non-Responders) repeated the cortisol assessment and *n* = 33 (*n* = 16 Responders and *n* = 17 Non-Responders) had cytokines measured in the serum. AUC, area under the curve.

### There Was no Difference Between Responders and Non-Responders in Recent and Early Life Stressors

A one-way ANOVA between Non-Responders, Responders, and healthy controls revealed significant differences among the 3 groups in number of recent stressful life events (*P* = .007), perceived stress (*P* < .001), and experience of childhood trauma (*P* < .001). However, when looking at post-hoc analyses for the 3 stress measurements, there were significant differences between the 2 patient groups and controls, but not between Responders and Non-Responders (see table 1).

### Exploratory Analysis of the Relationship Between Biomarkers of Treatment Response, and Symptom Severity and Clinical Improvement

Since we found that Non-Responders had more severe negative symptoms at baseline than Responders (see table 1), we explored the association between biomarkers of treatment response and negative symptoms at baseline. We did not find any significant association between baseline negative symptoms and either CAR (rho = 0.125, *P* = .4) or IL-6 levels (rho = −0.040, *P* = .8). However, we found a positive association between negative symptoms and IFN-γ levels (rho = 0.492, *P* = .004).

When we explored the association between percentage of clinical improvement and biomarkers of treatment response, we found that clinical improvement was significantly correlated with CAR (rho = 0.500, *P* = .003) and, at trend level, with IL-6 levels (rho = −0.300, *P* = .1), suggesting that lower CAR and higher IL-6 levels at baseline are associated with less improvement in clinical symptoms. In contrast we did not find a significant correlation between clinical improvement and baseline IFN-γ levels (rho = −0.097, *P* = .6).

However, in view of the fact that baseline IFN-γ levels were significantly associated with baseline PANSS negative symptoms, we explored whether baseline IFN-γ levels were also associated with severity of negative symptoms at follow-up. We found a significant positive correlation between baseline IFN-γ levels and severity of PANSS negative symptoms at follow-up (rho = 0.398, *P* = .03).

## Discussion

In this longitudinal study in first episode psychosis patients, we show for the first time that patients who subsequently do not respond to 12 weeks of treatment already have, at illness onset, a significant lower CAR and higher levels of IL-6 and IFN-γ, compared with patients who subsequently respond. Furthermore, differences in these biomarkers between Non-Responders and Responders persist over the first 12 weeks of treatment. Finally, comparison with controls demonstrate that these differences between Non-Responders and Responders are not qualitatively different from those present between patients and controls, but rather represent more severe biological abnormalities.

In this sample, we have found that the CAR at psychosis onset, but *not* diurnal cortisol levels, predicts subsequent treatment response. This is interesting, since we have found, in a previous sample partially overlapping with this one, that patients with first episode psychosis in general have a blunted CAR, together with high diurnal cortisol levels.^12^ However, the blunted CAR tends to remain unchanged with antipsychotic treatment, while the elevated diurnal cortisol levels tend to be normalized by antipsychotic treatment.^12,17^ Moreover, more blunted CAR (but *not* more elevated cortisol levels during the day) is associated with cognitive dysfunction in these patients.^16^ Therefore, it is possible that more blunted CAR is a biological “trait” marker, reflecting a more severe illness that cannot be modified by treatment. Interestingly, a recent study testing the effects of antiglucocorticoid treatment (mifepristone, RU486) on neuropsychological performance in patients with bipolar depression has shown that treatment with mifepristone is associated with an increase in CAR *and* with a sustained improvement in spatial working memory performance in these patients.^43^ Therefore, future studies should investigate the role of antiglucocorticoid treatments in ameliorating psychotic symptoms and improving cognitive function, especially in those patients showing a blunted CAR and thus less likely to respond to antipsychotic treatment. The fact that the CAR remains unchanged after 12 weeks of antipsychotic treatment in this study also confirms the notion that this is a trait marker.

Our findings also show that increased levels of inflammatory markers, in particular IL-6 and IFN-γ, are associated with poor treatment response in these patients. While increased inflammation in first episode psychosis has been described before,^19,44^ and previous studies have shown that higher inflammation (high levels of IL-2 and IL-6) is associated with a poorer clinical outcome in patients with treatment-resistant and/or chronic schizophrenia,^23,25,26^ our study is the first to show that this link is already present at the onset of psychosis. Furthermore, as per the CAR, the lack of changes over the 12 weeks for IL-6 indicated that this may also be a more trait-like marker. Of note, recent meta-analyses and reviews have reported mixed findings from studies testing the effects of adjunctive treatment with anti-inflammatory treatment in psychosis, with some agents, such as aspirin, showing some beneficial effects, and other agents, such as celecoxib and minocycline, showing no or limited effects.^45–47^ These conflicting findings may partly be explained by differences in length of treatment and patients’ selection,^47^ but particularly striking is the fact that none of the studies were stratified for baseline inflammation. As we have shown in this article, some patients have higher inflammation than others, and it is possible that only these are the patients who would truly benefit from an anti-inflammatory treatment. Consistent with this notion, a recent clinical trial with TNF-α antagonist in patients with treatment-resistant depression showed therapeutic effects only in the subsample of patients who had increased baseline inflammatory markers.^48^ Therefore, increased baseline inflammatory markers could be used to stratify patients in future trials evaluating the effectiveness of adjuvant anti-inflammatory treatment.

Surprisingly, we found an increase in IFN-γ levels over the 12 weeks in both Responders and Non-Responders, a finding that appears counterintuitive, since a recent meta-analysis report that antipsychotic treatment decreases this particular proinflammatory cytokine in patients.^49^ However, all of these studies assessed patients after a shorter interval (4–8 weeks), and it is possible that slightly longer treatment with atypical antipsychotics tends to induce production of cytokines through their propensity to generate metabolic syndrome.^23^

The mechanisms linking the blunted CAR and the increased inflammation with poor treatment response at the onset of psychosis could partly be explained by the well-known effects of cortisol and inflammation on monoaminergic pathways and on neuroplasticity.^2,41,50^ Notably, it has been recently suggested that the CAR prime the brain for the expected demands of the day,^51^ and that a blunted CAR predicts less neuroplasticity later in the afternoon, as shown by the response to rapid transcranial magnetic stimulation.^52^ Therefore, a blunted CAR in Non-Responders may be linked to a lower synaptic plasticity and to a suboptimal brain function, which might ultimately account for the inability of our patients to respond to treatment. As regards inflammation, previous studies have reported increased dopaminergic activity in various brain regions in offspring of rodents exposed to a prenatal inflammatory challenge.^53^ Furthermore, inflammatory processes negatively impact adult neurogenesis and contribute to wider neurodegenerative processes, through their influence on the kynurenine pathway and downstream production of the *N*-methyl-D-aspartate receptor agonist, quinolinic acid.^54^ The effect of inflammation on neuroplasticity at the onset of psychosis is further supported by our findings in first episode psychosis, showing an association between increased IL-6 levels and lower levels of brain-derived neurotrophic factor, which is a crucial mediator of adult neurogenesis and neuroplasticity.^3^

Interestingly, although patients had significantly higher levels of stress (ie, stressful life events, perceived stress, and experience of childhood trauma) when compared with controls, there were no significant differences between Responders and Non-Responders for any of these stress measures. Therefore, our findings suggest that high levels of stress in psychosis are not associated with poor treatment response per se; rather, they seem associated with the activation and reactivity of biological systems involved in the stress response, measured here as CAR and inflammatory markers, which can contribute to poor response or treatment resistance.

Finally, while CAR and IL-6 were also associated with overall clinical improvement, IFN-γ was more strongly associated with severity of negative symptoms both at baseline and at follow-up. These preliminary results suggest the presence of different molecular pathways associated with treatment response, with some inflammatory markers (in particular IFN-γ) being more associated to specific clinical profiles. However, our sample size was relatively small to test this particular hypothesis and, given the strong clinical heterogeneity of psychosis, we cannot exclude that the characteristics of our specific cohort may have contributed to this finding. Further studies would need to investigate this hypothesis in a larger and more powered sample.

Few limitations of the study need to be acknowledged. Firstly, this is a naturalistic study and we could not control the type and duration of antipsychotic treatment, which could have influenced the levels of stress biomarkers. However, there were no significant differences between Responders and Non-Responders in terms of duration and cumulative dose of treatment suggesting that antipsychotic treatment was not responsible for the difference in the stress biomarkers between the 2 groups. Secondly, most of our patients were already on antipsychotic treatment for 5–6 weeks when assessed at baseline, and therefore our findings might be more indicative of the very first few days/weeks of antipsychotic treatment rather than the time before starting any treatment. However, these findings could even be more interesting from a translational point of view, as they could aid the decision of clinicians to switch quickly to another and more effective medication in the early stages of antipsychotic treatment.

In conclusion, our findings show that blunted CAR and increased levels of proinflammatory cytokines predict poor treatment response at the onset of psychosis. These biomarkers hold strong potential as predictors of clinical outcome at the onset of psychosis as well as optimal targets for the development of novel therapeutic agents.

## Funding

This research has been supported by the National Institute for Health Research (NIHR) Mental Health Biomedical Research Centre at South London and Maudsley NHS Foundation Trust and King’s College London. This research has also been supported by a Starter Grant for Clinical Lecturers from the Academy of Medical Sciences to V.M.; and a grant from the Wellcome Trust (WT087417) to C.M.
